# Mediterranean Diet, Redox Biomarkers, and Body Composition in ICU Post-COVID-19 Patients

**DOI:** 10.3390/nu18152420

**Published:** 2026-07-24

**Authors:** Fernandes Daieni, Berbigier Marina Carvalho, Soares Cássia Medino, Spritzer Poli Mara, Marschner Rafael Aguiar, Wajner Simone Magagnin, Ferreira Samanta Catherine, Dall Alba Valesca

**Affiliations:** 1 Graduate Program: Sciences in Gastroenterology and Hepatology, Federal University of Rio Grande do Sul (UFRGS), Porto Alegre 90040-060, Brazil; daienifernandes77@gmail.com (F.D.); mcberbigier@hcpa.edu.br (B.M.C.); cassia.medino.cs@gmail.com (S.C.M.); 2 Graduate Program in Medical Sciences: Endocrinology and Metabolism, Federal University of Rio Grande do Sul, Porto Alegre 90040-060, Brazil; spritzer@ufrgs.br (S.P.M.); swajner@hcpa.edu.br (W.S.M.); 3 Department of Biochemistry, Federal University of Rio Grande do Sul, Porto Alegre 90040-060, Brazil; rafamarschner@gmail.com; 4 Graduate Program in Food, Nutrition, and Health, Federal University of Rio Grande do Sul, Porto Alegre 90040-060, Brazil; samanta.catherine@gmail.com; 5 Nutrition and Dietetics Service, Hospital de Clínicas de Porto Alegre, Porto Alegre 90040-060, Brazil

**Keywords:** Mediterranean diet, oxidative stress, redox status, body composition, inflammation, post-COVID-19 condition

## Abstract

**Background**: Post-COVID-19 condition (PCC) remains a public health concern due to persistent metabolic and inflammatory alterations, especially among ICU survivors. Oxidative stress is involved in PCC pathophysiology, and the Mediterranean diet may be associated with redox balance. **Objective**: To evaluate the association between Mediterranean diet adherence (main exposure variable) and oxidative stress/redox stress biomarkers (primary outcomes) in ICU survivors with persistent symptoms after severe COVID-19. Additionally, body composition and inflammatory markers were explored as secondary outcomes related to redox status. **Methods**: This cross-sectional study included 123 adult ICU survivors with PCC (51 ± 13.2 years; 53% men; 75% with obesity). Protein carbonyls, TBARS, sulfhydryl groups, glutathione, CRP, albumin, and IL-6 were measured. Diet adherence during post-COVID-19 outpatient follow-up was assessed using MEDAS, and body composition by DXA. Participants were categorized by the median score, and Poisson regression models were applied. **Results**: The median MEDAS total score was 4 (IQR 2–5), indicating overall low adherence to the Mediterranean diet. Lower adherence was associated with higher glutathione levels (*p* = 0.04), with no differences in pro-oxidant markers. Higher sulfhydryl levels were associated with lower body fat, higher appendicular skeletal muscle mass, and lower CRP and IL-6. **Conclusions**: Low adherence was frequent and associated with antioxidant differences. Redox status was also associated with body composition and inflammatory markers.

## 1. Introduction

The Coronavirus Disease 2019 (COVID-19) pandemic, caused by SARS-CoV-2, has had a profound impact on global health. Although the acute phase was characterized by high mortality, the medium- and long-term clinical consequences among survivors remain less understood, and follow-up data are still limited [1]. Post-COVID-19 condition (PCC), also referred to as long COVID or post-acute COVID-19 syndrome, is characterized by the persistence of symptoms not attributable to alternative diagnoses, lasting at least 12 weeks after acute infection, according to the World Health Organization (WHO) [2]. This syndrome represents an emerging challenge for patients, healthcare professionals, and public health systems, as its pathophysiological mechanisms and management strategies remain under debate.

Among the factors associated with PCC pathophysiology, oxidative stress (OS) plays a central role. Resulting from an imbalance between reactive oxygen species (ROS) and antioxidant defense mechanisms, OS induces redox dysfunction and promotes damage to proteins, lipids, and nucleic acids [3]. Viral infections frequently amplify this process by increasing ROS production in infected cells and activated phagocytes [4].

In this context, antioxidants, either endogenous or derived from the diet, play a protective role by neutralizing ROS or stimulating cellular antioxidant systems [5]. In addition to muscle loss associated with prolonged ICU stay, post-COVID-19 condition has been associated with persistent metabolic dysregulation, chronic low-grade inflammation, oxidative stress, reduced physical function, and altered adiposity distribution. Recent studies in post-ICU COVID-19 survivors have highlighted the clinical relevance of body composition and morphofunctional assessment during long-term recovery [6].

Nutrition is therefore a key modulator at the interface between oxidative stress and systemic inflammation. Dietary patterns rich in antioxidant compounds exert protective effects by attenuating OS-mediated cellular damage [7]. Among these, the Mediterranean diet (MedDiet) stands out for its high content of polyphenols, mono- and polyunsaturated fatty acids, and bioactive compounds with well-established antioxidant and anti-inflammatory properties. Evidence indicates that the MedDiet may modulate redox-inflammatory signaling pathways, such as Nrf2 and NF-κB, thereby positively influencing immunometabolic responses [7]. Moreover, recent systematic reviews suggest that greater adherence to the MedDiet is associated with a lower risk of COVID-19 infection and better clinical prognosis, although results remain inconsistent regarding its influence on persistent symptoms [8].

ICU survivors may represent a particularly vulnerable subgroup of patients with post-COVID-19 condition due to the combined effects of severe systemic inflammation, prolonged immobilization, muscle loss, and persistent metabolic and redox dysregulation associated with critical illness, as previously described by Davis et al. [9]. Despite advances in the understanding of COVID-19, an important knowledge gap remains regarding the effects of MedDiet adherence on long-term outcomes in post-COVID-19 condition, particularly in relation to oxidative stress, inflammation, and body composition. Therefore, the present study aimed to investigate the association between adherence to the Mediterranean diet, oxidative stress/redox biomarkers in ICU survivors with persistent symptoms after severe COVID-19. In addition, inflammatory parameters, and body composition were evaluated as secondary exploratory outcomes associated with redox status.

## 2. Materials and Methods

### 2.1. Study Design and Participants

This cross-sectional study included adults of both sexes diagnosed with post-COVID-19 condition, who were ICU survivors after severe COVID-19. All participants had been previously admitted to the intensive care unit of a public tertiary hospital between January and December 2021. Patients presenting persistent symptoms not explained by other clinical conditions for at least three months after hospital discharge were included. Individuals with cognitive impairment, pre-existing neurodegenerative diseases, and pregnant or lactating women were excluded.

Patients presenting persistent symptoms compatible with post-COVID-19 condition (PCC), according to World Health Organization criteria, defined as symptoms persisting for at least three months after acute SARS-CoV-2 infection and not explained by alternative diagnoses, were eligible for inclusion. Approximately three months after hospital discharge, ICU survivors were contacted by telephone and screened for persistent symptoms using a structured questionnaire. Participants reporting ongoing symptoms were invited for outpatient clinical evaluation, during which symptoms were reviewed by the research team and categorized according to body systems based on previous literature.

The study was conducted in accordance with the Declaration of Helsinki and approved by the institutional Research Ethics Committee (CAAE 2022-0215) in accordance with Resolution No. 466/12 of the National Health Council.

### 2.2. Clinical Assessment and Symptom Evaluation

Persistent symptoms were self-reported during outpatient visits using a structured questionnaire and categorized into ten body systems according to the literature. Information on pre-existing comorbidities and length of hospital stay was obtained from electronic medical records.

For descriptive purposes, the most prevalent symptom domains presented in Table 1 included musculoskeletal symptoms (e.g., myalgia, arthralgia, muscle weakness, and fatigue related to physical exertion), dermatologic symptoms (e.g., hair loss and cutaneous complaints), and immunologic symptoms (e.g., recurrent inflammatory or immune-related manifestations). These categories were used descriptively and were not intended to define diagnostic subtypes of post-COVID-19 condition.

Pre-existing comorbidities were identified from electronic medical records and confirmed during outpatient clinical evaluation whenever applicable. Obesity was defined as body mass index (BMI) ≥ 30 kg/m^2^. Hypertension, diabetes mellitus, chronic pulmonary disease, and cardiovascular disease were considered present when there was a previous documented medical diagnosis or current use of disease-specific medication. Chronic pulmonary disease included conditions such as asthma and chronic obstructive pulmonary disease (COPD), whereas cardiovascular disease included coronary artery disease, heart failure, arrhythmias, and other clinically documented cardiovascular conditions.

Sociodemographic variables, including educational level and place of residence, were collected during outpatient evaluation using a structured questionnaire administered by trained researchers. Educational level was classified according to the highest completed level of formal education.

### 2.3. Blood Sample Collection and Analysis

Fasting blood samples were collected on the day of clinical assessment. Serum was separated by centrifugation and stored at −80 °C until analysis.

### 2.4. Oxidative Stress Biomarkers

Protein carbonyls were determined according to Zanatta et al. [10]. Duplicate aliquots of serum homogenate (~0.3 mg protein) were incubated with 500 μL of 10 mM 2,4-dinitrophenylhydrazine plus 500 μL of 2 M HCl, or with 1.0 mL of 2 M HCl (blank tube). After 30 min, 250 μL of 50% trichloroacetic acid was added to the aliquots. Samples were then centrifuged at 8000× *g* for 30 min to obtain protein pellets, which were immediately washed with ethanol–ethyl acetate (1:1, *v*/*v*). The final protein pellets were resuspended in 500 μL of 8 M urea buffer and incubated at 50 °C for 90 min. The difference between samples treated with 2,4-dinitrophenylhydrazine and those treated with HCl (blank) was used to calculate carbonyl content, determined at 370 nm. Data were calculated using the hydrazine millimolar absorption coefficient (ε370 nm = 21,000 M^−1^·cm^−1^), and results were expressed as nmol carbonyl/mg protein.

Thiobarbituric acid reactive substances (TBARS) were analyzed according to Marschner et al. [11] as an indirect index of lipid peroxidation based on the method of Yagi [12], with minor modifications, and expressed as nmol/mg protein. Briefly, serum samples were incubated with thiobarbituric acid reagents, and the fluorescent MDA–TBA adduct was quantified fluorometrically using 1,1,3,3-tetramethoxypropane as standard.

Sulfhydryl groups were evaluated according to Aksenov and Markesbery [13]. Serum (~0.3 mg protein) was mixed with 5-thio-2-nitrobenzoic acid (TNB), derived from the reaction of thiol groups with 5,5′-dithiobis-(2-nitrobenzoic acid) (DTNB), forming a yellow-colored derivative measured spectrophotometrically at 412 nm. Results were expressed as nmol TNB/mg protein. and expressed as nmol TNB/mg protein.

Reduced glutathione (GSH) was quantified according to Browne and Armstrong [14]. Briefly, 185 μL of 100 mM sodium phosphate buffer (pH 8.0) containing 5 mM EDTA and 15 μL of o-phthalaldehyde solution (1 mg/mL) were added to 30 μL of serum (~0.3–0.5 mg protein) previously deproteinized with metaphosphoric acid. The mixture was incubated at room temperature in a dark room for 15 min. Fluorescence was measured using excitation and emission wavelengths of 350 and 420 nm, respectively. A calibration curve was prepared using standard GSH (0.001–1 mM), and concentrations were calculated as nmol GSH/mg protein.

### 2.5. Inflammatory Markers

IL-6 was measured using ultrasensitive ELISA (25% dilution). C-reactive protein (CRP) was measured by immunoturbidimetry (Cobas c702). Albumin was analyzed using the bromocresol green colorimetric method (Cobas c702).

The selection of oxidative stress and inflammatory biomarkers was designed to capture complementary aspects of the redox-inflammatory axis. Markers of oxidative damage (TBARS and protein carbonyls), antioxidant defense (GSH and sulfhydryl groups), and systemic inflammation (IL-6, CRP, and albumin) were included to provide an integrated assessment of redox homeostasis and inflammatory status, both of which are central to the pathophysiology of post-COVID-19 condition.

### 2.6. Mediterranean Diet Adherence

Adherence to the MedDiet was assessed during the outpatient clinical evaluation performed after ICU discharge using the Mediterranean Diet Adherence Screener (MEDAS), validated for Brazilian Portuguese. Therefore, the questionnaire reflected participants’ current dietary habits during the post-COVID-19 recovery period rather than dietary patterns before SARS-CoV-2 infection or hospitalization. The questionnaire includes 14 items scored as 1 (adherent) or 0 (non-adherent). Although scores ≥ 10 indicate good adherence, the median score was used as the cutoff due to the low overall adherence observed in ICU survivors.

All questionnaires were administered during face-to-face outpatient evaluations by trained researchers, allowing clarification of questions and confirmation of responses when necessary.

### 2.7. Body Composition Assessment

Body composition was assessed by dual-energy X-ray absorptiometry (DXA) (GE Lunar Prodigy^®^ GE Healthcare, Madison, WI, USA) Total and regional fat mass, lean mass, and appendicular skeletal muscle mass were obtained. DXA was performed within seven days of the initial clinical evaluation.

### 2.8. Statistical Analysis

The primary outcomes of this study were oxidative stress/redox biomarkers (GSH, sulfhydryl groups, TBARS, and protein carbonyls). Mediterranean diet adherence assessed by MEDAS was considered the primary exposure variable. Body composition and inflammatory markers were analyzed as secondary exploratory variables.

Normality was assessed using the Shapiro–Wilk test. Continuous variables were expressed as mean ± standard deviation or median (interquartile range), as appropriate. Categorical variables were expressed as absolute and relative frequencies. Group comparisons were performed using Student’s *t*-test or Mann–Whitney test for continuous variables and chi-square or Fisher’s exact test for categorical variables.

Two Poisson regression models with robust variance were applied. The first model evaluated associations between MedDiet adherence and oxidative stress biomarkers, adjusted for sex, age, physical activity level, and obesity as a priori confounders. The second model investigated associations between body composition parameters, inflammatory markers, and number of persistent PCC symptoms with oxidative stress biomarkers. Statistical significance was set at *p* < 0.05. Analyses were conducted using SPSS^®^ Statistics v.27.

## 3. Results

In 2021, a total of 1049 patients were admitted to the ICU of a university hospital in Southern Brazil due to COVID-19; among them, 440 (41.9%) died. Of the 588 survivors, 317 were contacted by telephone three months after hospital discharge, and 252 met the criteria for post-COVID condition (PCC). Ultimately, 123 patients agreed to participate and were effectively included in the study. Exclusions occurred due to refusal or inability to establish contact.

**Figure 1:**
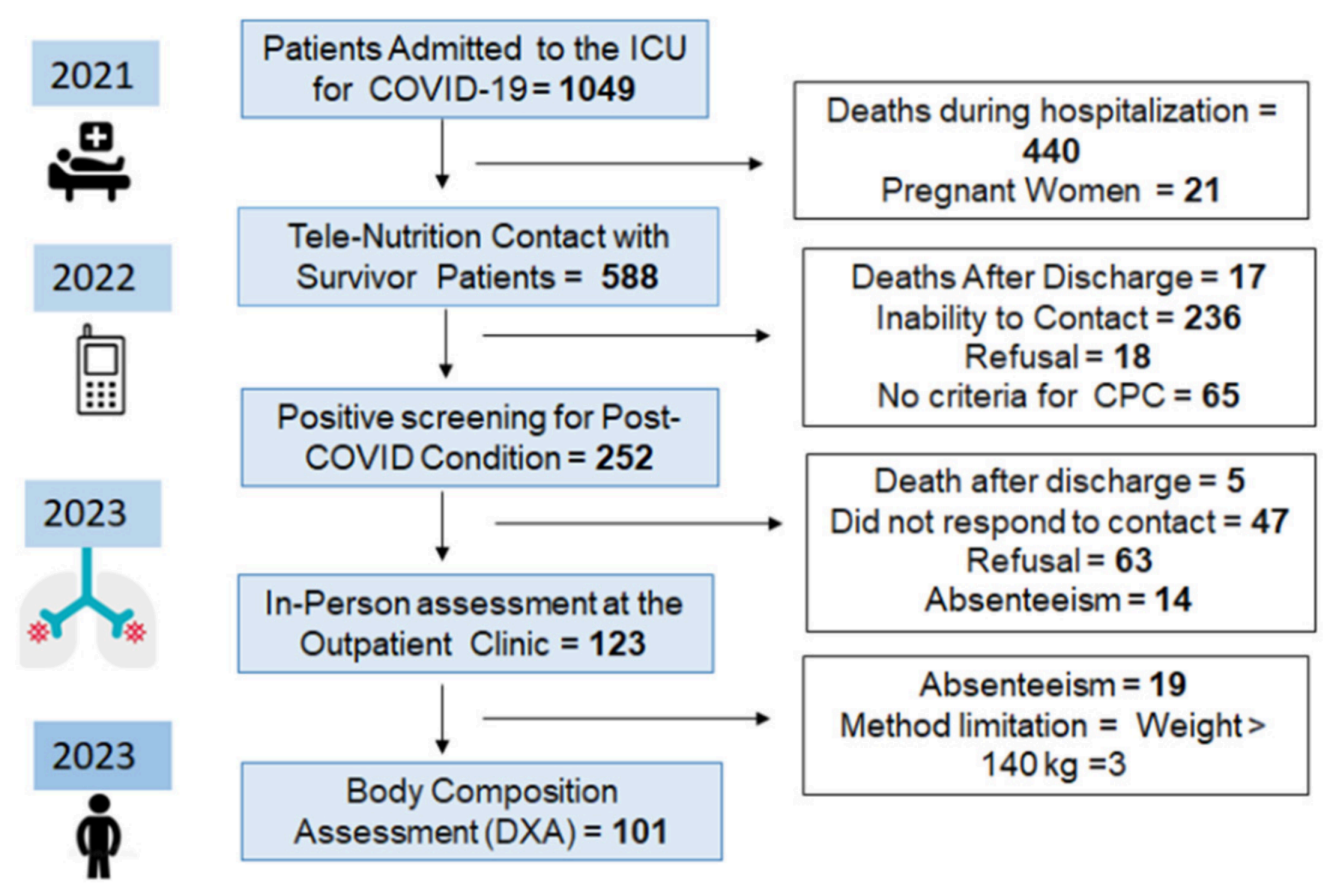
Flowchart of patient selection and inclusion. Abbreviation: PCC, post–COVID-19 condition.

Figure 1 presents the flowchart of patient selection and inclusion.

Table 1 summarizes the demographic, clinical, nutritional, and biochemical characteristics of the participants. Overall, 82.1% of patients self-identified as White, 48.5% had completed high school education, and 69.1% resided in the state capital. At three months post-discharge, all patients (100%) reported persistent symptoms.

**Figure 2:**
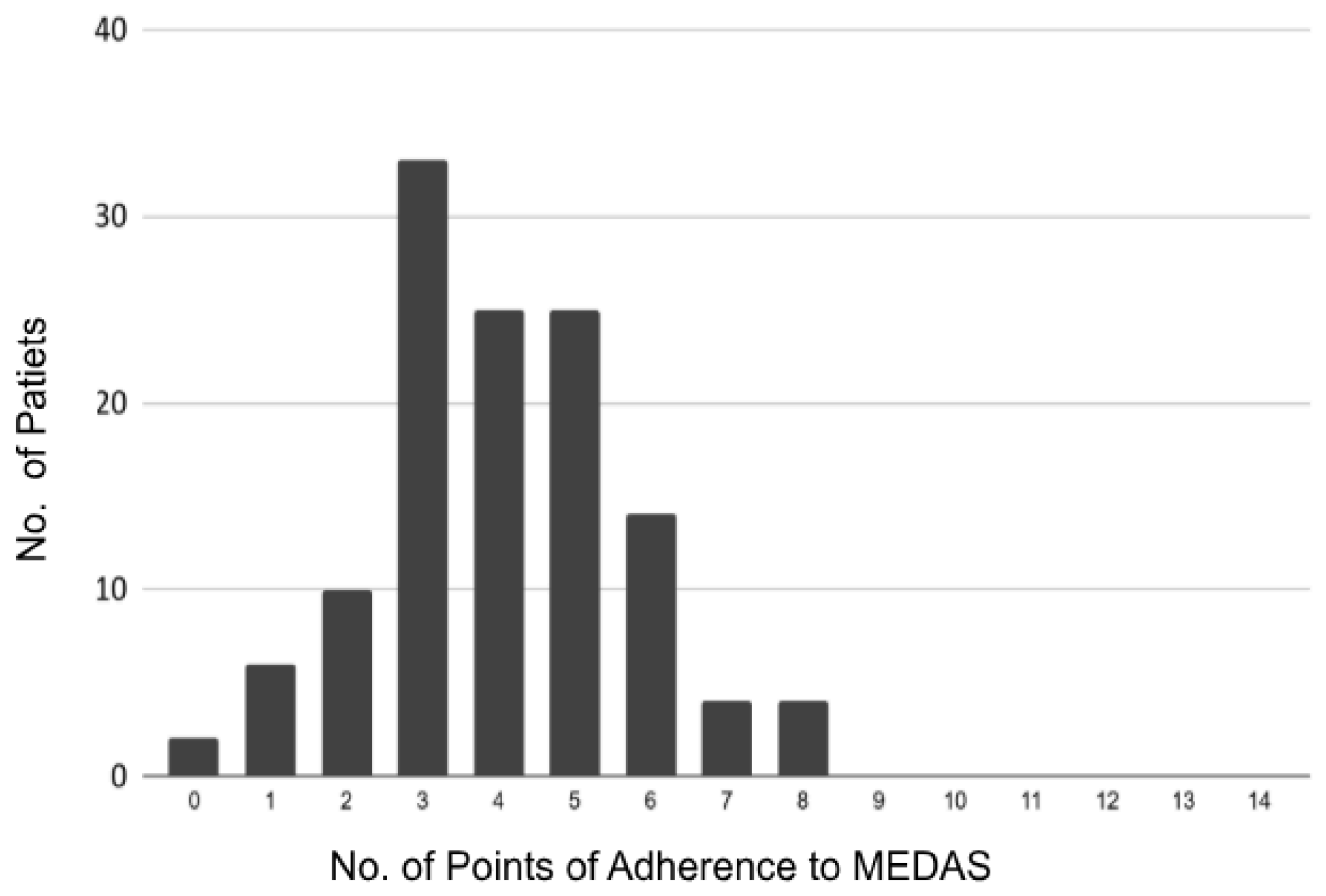
Distribution of participants according to total score on the Mediterranean Diet Adherence Screener (MEDAS). Abbreviation: MEDAS, Mediterranean Diet Adherence Screener.

Figure 2 shows the distribution of participants by total MEDAS score (median = 4).

The median score was 4 points, indicating that half of the sample scored ≤ 4.

In Table 2, oxidative stress biomarkers were categorized according to the median MEDAS score into two groups: lower adherence (score ≤ 4) and higher adherence (score > 4). Participants with lower adherence to the Mediterranean diet (MEDAS ≤ 4) presented higher levels of GSH (*p* < 0.04). Those with higher adherence showed lower levels of pro-oxidant markers (TBARS and protein carbonyls), although these differences did not reach statistical significance.

Table 3 describes inflammatory, body composition, and clinical markers categorized according to the median plasma concentration of sulfhydryl groups. Higher sulfhydryl levels were significantly associated with lower body fat percentage (*p* = 0.02), greater appendicular muscle mass (*p* < 0.01), lower IL-6 levels (*p* < 0.01), lower C-reactive protein concentrations (*p* = 0.04), and higher albumin levels (*p* < 0.01). No significant association was observed between sulfhydryl levels and the number of persistent symptoms (*p* = 0.18).

No significant differences were found between groups categorized by the median values of GSH, protein carbonyls, or TBARS with respect to biochemical, body composition, or clinical variables.

Sulfhydryl groups were selected for further stratified analyses due to their role as a stable and integrative marker of systemic redox status, reflecting the overall antioxidant buffering capacity of circulating proteins. In addition, among the redox biomarkers evaluated, sulfhydryl levels showed the most consistent associations with both inflammatory and body composition parameters. Therefore, this marker was used as a central variable to further explore the interplay between redox balance, inflammation, and metabolic status in post-COVID-19 condition.

Higher sulfhydryl concentrations were significantly associated with lower body fat percentage, greater appendicular skeletal muscle mass, lower IL-6 and CRP concentrations, and higher albumin levels.

## 4. Discussion

In the present study involving ICU survivors with post-COVID condition, patients with lower adherence to the Mediterranean diet exhibited higher levels of reduced glutathione (GSH), an essential endogenous antioxidant responsible for neutralizing reactive oxygen species (ROS) and maintaining redox homeostasis. Reduced glutathione has been described as a central marker in the modulation of oxidative stress (OS), and its depletion may exacerbate inflammatory processes and increase susceptibility to complications associated with SARS-CoV-2 infection, including post-COVID condition [15] The elevated GSH levels observed in individuals with lower dietary adherence may reflect a compensatory adaptive response to the persistent oxidative stress characteristic of PCC in ICU survivors, suggesting upregulation of endogenous antioxidant defense mechanisms. Importantly, Mediterranean diet adherence was assessed during outpatient follow-up after ICU discharge. Therefore, the MEDAS score reflects the participants’ current dietary pattern during recovery from post-COVID-19 condition rather than dietary habits before SARS-CoV-2 infection or during the acute phase of illness. Thus, the observed associations should be interpreted as cross-sectional relationships and not as evidence of causality or prior protective effects.

In a study involving 600 patients with COVID-19, Mohajeri et al. [16] reported that greater adherence to the Mediterranean diet was associated with lower TBARS levels and higher total antioxidant capacity. In our sample, although TBARS levels were lower among individuals with higher Mediterranean diet adherence, the difference did not reach statistical significance (*p* = 0.06). This observed pattern may represent a biologically plausible effect limited by sample size, interindividual variability, and the prolonged interval since the acute phase of infection. Although statistical significance was not achieved, our findings demonstrate a directionally consistent association with the hypothesis that the Mediterranean diet may exert protective effects against lipid peroxidation and contribute to redox balance modulation in ICU survivors, in agreement with previous literature [8,15].

Another relevant finding was the association between higher sulfhydryl concentrations and a more favorable body composition profile, characterized by lower body fat percentage, greater appendicular muscle mass, and reduced inflammatory markers (CRP and IL-6). These results are supported by Zhang et al. [17], who reported a positive correlation between total antioxidant capacity and lean mass, and a negative association with BMI and body fat.

This finding is particularly relevant in ICU survivors, who frequently present persistent muscle loss, metabolic dysregulation, and prolonged inflammatory activation following critical illness. The relationship observed in our study may be partially explained by pathophysiological mechanisms associated with excess adiposity, which increases free fatty acid release and activates NADPH oxidase in adipocytes and peripheral tissues, thereby amplifying ROS production and promoting chronic inflammation [18].

In addition to dietary factors, physical rehabilitation and structured exercise interventions may also play an important role in modulating redox balance and improving body composition in post-COVID-19 ICU survivors. Exercise has been associated with enhanced mitochondrial function, reduced oxidative stress, and attenuation of chronic low-grade inflammation. Given that ICU survivors frequently present with muscle loss and metabolic impairment, the combination of exercise and adherence to antioxidant-rich dietary patterns, such as the Mediterranean diet, may exert complementary effects supporting functional recovery and metabolic health. This integrated approach may be particularly relevant for long-term recovery in post-COVID-19 condition, as previously discussed by Barrea et al. [19].

Recent evidence also suggests that gut microbiota alterations following ICU hospitalization and SARS-CoV-2 infection may contribute to persistent inflammation, immune dysregulation, and oxidative stress during post-COVID-19 recovery through gut–immune and gut–lung axis interactions. In this context, dietary patterns such as the Mediterranean diet may modulate gut microbiota diversity and the production of anti-inflammatory metabolites, potentially influencing metabolic and redox recovery in ICU survivors [20].

Within this context, adherence to the Mediterranean diet may exert protective effects on body composition, particularly regarding muscle mass preservation, through reduction in systemic oxidative burden. Higher intake of dietary antioxidants contributes to ROS neutralization in skeletal muscle, attenuating oxidative stress and protein degradation. Additionally, micronutrients such as magnesium and selenium, associated with greater adherence to this dietary pattern, play roles in muscle energy production and in the activity of antioxidant enzymes such as glutathione peroxidase, supporting myocyte protection against oxidative damage [21]. These mechanisms provide biological plausibility for the observed association between higher sulfhydryl levels, lower systemic inflammation, and improved body composition.

No significant association was observed between GSH levels and body composition parameters. This finding may reflect methodological limitations, as GSH measurement is highly susceptible to pre-analytical variability and rapid oxidation during sample processing, potentially leading to underestimation of actual concentrations. Furthermore, plasma GSH levels do not necessarily reflect tissue compartments more directly related to body composition, such as skeletal muscle and liver. This is consistent with evidence indicating that pro-oxidant markers such as carbonyls and TBARS do not always directly reflect oxidative burden in chronic conditions, as repair and clearance processes of modified biomolecules may attenuate detectable levels [22].

From a dietary perspective, we identified low adherence to the Mediterranean diet in our sample (median MEDAS score of 4), reflecting a pattern widely documented in different populations. Studies indicate that adherence to the Mediterranean diet tends to be lower outside the Mediterranean region and, even within Mediterranean countries, scores have ranged from low to moderate in recent years [8,23].

Explanatory factors include cultural barriers, local dietary preferences, higher costs, and limited availability of key foods such as olive oil, fish, fresh fruits, and nuts [24].

This scenario was likely exacerbated during the pandemic due to food insecurity, economic crises, and disruptions in supply chains. According to Ammar et al. [25] lockdown periods were associated with reduced diet quality, characterized by increased consumption of ultra-processed foods and lower intake of fruits and vegetables. Therefore, ICU survivors with PCC may have faced additional obstacles in maintaining protective dietary patterns during the recovery period.

Educational level is another relevant factor: only 9.8% of our sample had completed higher education. Evidence suggests that higher educational attainment is associated with better adherence to healthy dietary patterns, including the Mediterranean diet [23]. Thus, beyond economic barriers, sociocultural determinants likely contributed to the low MEDAS scores observed.

The low MEDAS score reflects insufficient adherence to the Mediterranean diet and consequently reduced intake of polyphenols, flavonoids, and monounsaturated fatty acids. This dietary limitation may increase vulnerability to persistent oxidative stress and contribute to the maintenance of chronic inflammatory processes during the post-infection period. Previous intervention studies have also demonstrated that adherence to the Mediterranean diet may increase circulating bioflavonoid levels and reduce inflammatory markers [26]. Recent reviews suggest that patients with PCC present nutritional imbalances that may perpetuate symptoms and delay functional recovery [19,27]. Our findings suggest that greater adherence to the Mediterranean diet could attenuate these imbalances through modulation of oxidative stress and inflammation, consistent with evidence that this dietary pattern is associated with improvements in body composition and, indirectly, quality of life [28].

Given the cross-sectional nature of this study, the associations observed between Mediterranean diet adherence, redox biomarkers, inflammation, and body composition should be interpreted as observational findings rather than causal relationships. Thus, it cannot be determined whether pre-existing metabolic and oxidative alterations increased susceptibility to severe COVID-19 and persistent symptoms, or whether post-COVID-19 condition itself contributed to sustained metabolic and redox disturbances during recovery.

The literature also describes complementary approaches, such as supplementation with GSH precursors (e.g., N-acetylcysteine), which may enhance antioxidant defense [29]. However, practical implementation of the Mediterranean diet in Brazil requires cultural and socioeconomic adaptations [30]. The Brazilian Dietary Guidelines represent an important strategy in this regard, promoting the consumption of unprocessed and minimally processed foods, home cooking, and shared meals—principles aligned with the Mediterranean dietary pattern [31].

Therefore, public policies promoting access to fresh foods and nutritional education initiatives may be essential to improve adherence and reduce the risk of perpetuating metabolic and inflammatory disturbances in vulnerable ICU survivor populations.

This study has limitations, including its cross-sectional design, which precludes causal inference, and its single-center setting involving previously hospitalized severe COVID-19 ICU survivors from Southern Brazil, where regional dietary habits, socioeconomic characteristics, and cultural factors may differ from other populations, potentially limiting the generalizability of the findings.

Sample size may also have reduced statistical power, particularly for variables with high interindividual variability. To mitigate these effects, we applied well-defined inclusion criteria, used standardized laboratory methods, and adjusted analyses for potential confounders. These measures enhance the reliability of the findings and suggest that, despite limitations, the results contribute meaningfully to the understanding of PCC in ICU survivors.

On the other hand, important strengths should be highlighted: this is one of the first studies to evaluate the association between Mediterranean diet adherence and oxidative stress biomarkers in ICU survivors with PCC, using DXA—considered the gold standard for body composition assessment—alongside multiple inflammatory and antioxidant markers. Another relevant aspect was the extended follow-up period (17 months after hospital discharge), allowing observation of sustained post-infection effects in critically ill survivors. Finally, patients received individualized feedback and practical guidance, reinforcing the clinical applicability of the findings.

## 5. Conclusions

Low adherence to the Mediterranean diet was associated with alterations in redox balance in ICU survivors with post-COVID-19 condition, evidenced by higher glutathione levels, which may reflect a compensatory response to persistent oxidative stress rather than a more favorable antioxidant status. Additionally, higher sulfhydryl concentrations were associated with a more favorable body composition and lower systemic inflammation, even 17 months after hospital discharge. These findings support the hypothesis that the Mediterranean diet, recognized for its antioxidant and anti-inflammatory potential, may be associated with metabolic recovery after severe COVID-19. Longitudinal and multicenter studies with larger samples are needed to confirm these associations and explore the applicability of the Mediterranean diet as an adjunct nutritional strategy in the management of post-COVID-19 condition.

## Figures and Tables

**Table 1:** Demographic, clinical, nutritional, and biochemical characteristics of patients with post–COVID-19 condition (n = 123).

Variables	Values
Age, years (mean ± SD)	51.7 ± 13.2
Sex, n (%) Male	66 (53.7)
Preexisting comorbidities, n (%)	
Obesity	92 (74.9)
Hypertension	49 (39.8)
Chronic pulmonary disease	21 (17.1)
Diabetes mellitus	33 (26.8)
Cardiovascular disease	17 (13.8)
Persistent symptoms after 17 months, n (%)	111 (90.2)
Number of persistent post–COVID-19 symptoms, median (IQR)	3 (2–5)
Most prevalent symptom categories, n (%)	
Musculoskeletal	78 (63.4)
Dermatologic	71 (57.7)
Immunologic	66 (53.7)
Body mass index (BMI), kg/m^2^, median (IQR)	33.9 (20–72)
Body composition	
Fat mass, % (mean ± SD)	42.9 ± 9.1
Fat mass, kg (mean ± SD)	39.4 ± 13.0
Appendicular skeletal muscle mass, kg, median (IQR)	22.9 (18–29.2)
Total lean mass, kg (mean ± SD)	52 ± 11
Mediterranean Diet Adherence Screener (MEDAS), median (IQR)	4 (2–5)
Oxidative stress biomarkers, median (IQR)	
Reduced glutathione (GSH), nmol/mg protein	0.6 (0.5–0.8)
Thiobarbituric acid reactive substances (TBARS), nmol/mg protein	75.1 (56.1–90.6)
Sulfhydryl content, nmol/mg protein	3.1 (1.2–9.5)
Protein carbonyl content, nmol/mg protein	13.7 (9.6–18.3)
Inflammatory markers, median (IQR)	
Albumin, mg/Dl	4.3 ± 0.3
C-reactive protein (CRP), mg/dL	4.5 (2–11.9)

Values are expressed as mean ± SD or median (IQR), as appropriate. Abbreviations: BMI, body mass index; CRP, C-reactive protein; GSH, reduced glutathione; IQR, interquartile range; MEDAS, Mediterranean Diet Adherence Screener; TBARS, thiobarbituric acid reactive substances.

**Table 2:** Frequency of patients with adherence to the Mediterranean Diet according to oxidative stress biomarker levels.

Variables	Higher GSH, n (%)	Higher Sulfhydryl, n (%)	Lower TBARS, n (%)	Lower Carbonyls, n (%)
MEDAS > 4	18 (39)	19 (41)	29 (63)	25 (54)
MEDAS ≤ 4	42 (56)	41 (55)	32 (43)	36 (48)
*p*-value	0.04	0.58	0.06	0.50

Abbreviations: GSH, reduced glutathione; TBARS, thiobarbituric acid reactive substances; MEDAS, Mediterranean Diet Adherence Screener. Notes: Higher GSH (>median 0.67 nmol/mg), higher sulfhydryl (>median 3.10 nmol/mg), lower TBARS (<median 75.11 nmol/mg), lower carbonyls (<median 13.74 nmol/mg). Chi-square or Fisher’s exact test; analyses adjusted for age, sex, physical activity, and obesity. Significance at *p* < 0.05.

**Table 3:** Biochemical, body composition, and clinical parameters categorized according to the median plasma concentration of sulfhydryl groups.

Variables	Sulfhydryl ≤ 3.10	Sulfhydryl > 3.10	*p*-Value
Body composition			
DXA body fat, % (mean ± SD)	46.22 ± 1.20	40.05 ± 1.23	0.02
DXA appendicular muscle mass, kg, median (IQR)	19.81 (6.92–25.52)	27.30 (21.47–31.70)	0.01
Inflammatory markers			
Interleukin-6 (IL-6), pg/mL, median (IQR)	0.86 (0.00–3.01)	0.00 (0.00–1.49)	0.01
C-reactive protein (CRP), mg/dL, median (IQR)	4.80 (2.50–17.50)	3.25 (1.45–8.25)	0.04
Albumin, mg/dL, median (IQR)	4.30 (3.30–4.90)	4.50 (3.80–5.20)	0.01
Clinical Variable			
Number of persistent symptoms, median (IQR)	4 (2–6)	2.5 (1.00–4.00)	0.18

Abbreviations: DXA, dual-energy X-ray absorptiometry; IL-6, interleukin-6; CRP, C-reactive protein. Data are expressed as median (interquartile range) or mean ± SD. Student’s *t* test or Mann–Whitney U test. Analyses adjusted for age, sex, physical activity, and obesity. Significance at *p* < 0.05.

## Data Availability

No new datasets were created or analyzed during the current study.

## Abbreviations

COVID-19	Coronavirus disease 2019
SARS-CoV-	2 Severe acute respiratory syndrome coronavirus
PCC	Post-COVID-19 condition
OS	Oxidative stress
ROS	Reactive oxygen species
MedDiet	Mediterranean diet
TBARS	Thiobarbituric acid reactive substances
GSH	Reduced glutathione
MEDAS	Mediterranean diet adherence screener
DXA	Dual-energy X-ray absorptiometry
BMI	Body mass index
CRP	C-reactive protein
IL-6	Interleukin-6
